# Assessment of hearing threshold in adults with hearing loss using an automated system of cortical auditory evoked potential detection^☆^

**DOI:** 10.1016/j.bjorl.2016.02.016

**Published:** 2016-04-29

**Authors:** Alessandra Spada Durante, Margarita Bernal Wieselberg, Nayara Roque, Sheila Carvalho, Beatriz Pucci, Nicolly Gudayol, Kátia de Almeida

**Affiliations:** Faculdade de Ciências Médicas da Santa Casa de São Paulo, São Paulo, SP, Brazil

**Keywords:** Auditory evoked potentials, Auditory perception, Hearing aids, Hearing loss, Electrophysiology, Potenciais evocados auditivos, Percepção auditiva, Auxiliares de audição, Perda auditiva, Eletrofisiologia

## Abstract

**Introduction:**

The use of hearing aids by individuals with hearing loss brings a better quality of life. Access to and benefit from these devices may be compromised in patients who present difficulties or limitations in traditional behavioral audiological evaluation, such as newborns and small children, individuals with auditory neuropathy spectrum, autism, and intellectual deficits, and in adults and the elderly with dementia. These populations (or individuals) are unable to undergo a behavioral assessment, and generate a growing demand for objective methods to assess hearing. Cortical auditory evoked potentials have been used for decades to estimate hearing thresholds. Current technological advances have lead to the development of equipment that allows their clinical use, with features that enable greater accuracy, sensitivity, and specificity, and the possibility of automated detection, analysis, and recording of cortical responses.

**Objective:**

To determine and correlate behavioral auditory thresholds with cortical auditory thresholds obtained from an automated response analysis technique.

**Methods:**

The study included 52 adults, divided into two groups: 21 adults with moderate to severe hearing loss (study group); and 31 adults with normal hearing (control group). An automated system of detection, analysis, and recording of cortical responses (HEARLab^®^) was used to record the behavioral and cortical thresholds. The subjects remained awake in an acoustically treated environment. Altogether, 150 tone bursts at 500, 1000, 2000, and 4000 Hz were presented through insert earphones in descending-ascending intensity. The lowest level at which the subject detected the sound stimulus was defined as the behavioral (hearing) threshold (BT). The lowest level at which a cortical response was observed was defined as the cortical electrophysiological threshold. These two responses were correlated using linear regression.

**Results:**

The cortical electrophysiological threshold was, on average, 7.8 dB higher than the behavioral for the group with hearing loss and, on average, 14.5 dB higher for the group without hearing loss for all studied frequencies.

**Conclusion:**

The cortical electrophysiological thresholds obtained with the use of an automated response detection system were highly correlated with behavioral thresholds in the group of individuals with hearing loss.

## Resumo

**Introdução:**

A utilização da amplificação sonora por pessoas com perda auditiva oferece uma melhor qualidade de vida. O acesso a este recurso e o seu benefício podem ficar comprometidos no caso de pacientes que apresentem dificuldades ou limitações na avaliação audiológica tradicional comportamental, tais como neonatos e crianças pequenas, presença do espectro da neuropatia auditiva e do autismo, déficit intelectual e presença de estados demenciais de adultos e idosos. Estas populações (ou indivíduos) incapazes de participar de uma avaliação comportamental geram uma crescente demanda por métodos objetivos de avaliação auditiva. Os potenciais evocados auditivos corticais são utilizados há decadas, com a finalidade de estimar os limiares auditivos. Avanços tecnológicos atuais permitiram o desenvolvimento de equipamentos que possibilitam seu uso clínico, dotados de recursos que permitem maior precisão, sensibilidade e especificidade, além da possibilidade de detecção, análise e registro automatizados das respostas corticais.

**Objetivo:**

Determinar e correlacionar o limiar auditivo comportamental com o limiar auditivo cortical obtidos em equipamento de análise automatizada das respostas.

**Método:**

Participaram do estudo 52 adultos, distribuídos em dois grupos: 21 adultos com perda de grau moderado a severo (Grupo Estudo), e 31 com audição normal (Grupo Controle). Para o registro dos limiares comportamentais e corticais foi utilizado um equipamento dotado de um sistema com detecção, análise e registro automatizados das respostas corticais (HEARLab^®^). Os participantes permaneceram despertos, em um ambiente acusticamente tratado. Foram apresentados 150 estímulos tipo *tone burst* nas frequências de 500, 1.000, 2.000 e 4.000 Hz, por meio de fones de inserção em intensidades descendente-ascendente. O menor nível no qual o sujeito detectou a presença do estímulo sonoro foi definido como o limiar auditivo comportamental. O menor nível no qual uma resposta cortical estava presente foi definido como o limiar eletrofisiológico cortical. Essas duas respostas foram correlacionadas por meio da regressão linear.

**Resultados:**

O limiar eletrofisiológico cortical foi, em média, 7,8 dB superior ao comportamental para o grupo com perda auditiva e 14,5 dB superior, em média, para o grupo sem perda auditiva para todas as frequências estudadas.

**Conclusão:**

Os limiares eletrofisiológicos corticais obtidos por meio de um sistema de detecção automatizado de respostas estavam fortemente correlacionados com os limiares comportamentais no grupo de indivíduos com perda auditiva.

## Introduction

The use of hearing aids by individuals with hearing loss brings a better quality of life. Access to and benefit from these devices may be compromised in patients with difficulties or limitations in traditional behavioral audiological evaluation, such as newborns and small children, individuals with auditory neuropathy spectrum, autism, intellectual deficits, and in adults and the elderly with dementia. These populations (or individuals) are unable to undergo a behavioral assessment, and generate a growing demand for objective methods to assess hearing.

Cortical auditory evoked potentials (CAEP) have been the focus of interest and study since the 1960s and 1970s. In the past, the principal application of this potential was an objective estimation of hearing threshold in difficult-to-test adults, but it was also extensively investigated in children.^1^

The assessment of hearing thresholds using CAEP has numerous advantages as it assesses the entire auditory system from brainstem to cortex. It can be recorded in conscious subjects using a variety of acoustic stimuli presented either through earphones or in open field.^2^, ^3^, ^4^, ^5^ Although it has inestimable scientific and clinical value, the routine use of these cortical potentials has been hindered over the last years by numerous factors. The main CAEP components undergo substantial changes in the response pattern depending on the stage of development from birth to adolescence, as well as when the evaluation is performed during intermediate stages of drowsiness. There is also variability in response amplitude, latency, and morphology both within and between subjects. These variabilities provide difficulties in recognition and interpretation of the responses, which require experienced and specialized professionals. In addition to these factors, the high cost of equipment has added to the technical limitation of the electrodes, filters, and amplifiers necessary to capture these potentials, and until recently minimized the clinical use of CAEP.^3^, ^6^

In order to overcome these barriers and promote its clinical use, the National Acoustic Laboratory (NAL), an Australian government institution, over the past few years has developed a device for the investigation of cortical auditory evoked potentials named HEARLab^®^ (Frye Electronics; United States). The difference of this system to its currently available counterparts is that it is more affordable and has the potential for clinical use. Among other features, it contains advanced and differentiated technologies that can reduce the registration of noise and interference by providing residual noise measurements, and has electrodes that are more sensitive in capturing responses. However, the principal difference is that it relies on a unique method of automatic detection and analysis of responses that takes into account statistical methods and tests, similar to the *t*-test, that determines the presence or absence of cortical response through confidence level calculations. This software exempts the examiner from the difficult task of subjectively interpreting the presence or absence of cortical response based solely on a visual analysis.^1^, ^3^, ^5^, ^7^, ^8^, ^9^, ^10^, ^11^, ^12^, ^13^, ^14^

The hypothesis of this study is that it is possible to estimate the behavioral hearing thresholds based on the cortical electrophysiological thresholds obtained from automatic analysis equipment.

In Brazil, there no studies of auditory thresholds have been performed with an automated analysis equipment of cortical response. The present study aimed to analyze the use of CAEP to estimate hearing thresholds through an automated CAEP response analysis equipment.

## Methods

This study was approved by the Research Ethics Committee of the institution under No. 361/11. All participants were informed about the objectives of the study and signed an informed consent form.

### Case series

The following inclusion criteria were established:

• Group C (control): adults (age ≤ 65 years), of both sexes, with normal hearing (tritonal mean of 500, 1000, and 2000 Hz <20 dB HL), without hearing complaints or history of otologic problems.
• Group S (study): adults (age ≤ 65 years), of both sexes, with bilateral symmetrical sensorineural hearing loss that was moderate to severe (tritonal means of 500, 1000, and 2000 Hz ≥41 dB HL and ≤90 dB HL).^15^

The exclusion criteria were the presence of neurological, psychiatric impairment, and/or declared or proven syndromes.

### Procedures

All sample subjects underwent the same evaluation protocol specified below.

To rule out problems in the middle ear that would prevent the inclusion in the study, visual inspection and tympanometry were performed using a middle ear analyzer (Interacoustics Model AZ-7R).

For the investigation of behavioral pure tone thresholds, the audiometer GN Otometrics Itera was used with supra-aural TDH-39 earphones in acoustic booths. Full behavioral audiometric evaluation was performed by pure tones at frequencies 250–8000 Hz for air and 500–4000 Hz for bone conduction using the descending–ascending classical technique of tonal threshold assessment. These thresholds were herein termed audiometric thresholds (AT).

For the assessment of behavioral hearing thresholds for tone burst stimulus, the HEARLab^®^ system was used. The equipment provides auditory stimuli at 500, 1000, 2000, 3000, and 4000 Hz, at intensities ranging from 0 to 110 dB HL, presented through insert earphones in an acoustically treated room. The descending–ascending threshold measurement technique was used, and the lowest intensity at which the subject was able to detect the tone burst presented was termed behavioral threshold (BT).

In preparation for the CAEP assessment, the patient's skin was properly cleaned and prepared. The electrodes were placed according to the following position: active electrode in the vertex (Cz), reference electrode in the right or left mastoid (M1 and M2), ground electrode in the forehead (Fz); the minimum acceptable conditions of maximum impedance and between electrodes (≤5 kΩ) were ensured. Participants were assessed while awake, distracted by images aired on a TV without sound, in an acclimatized and acoustically treated room.

Assessment of BT and cortical electrophysiological threshold (CET) at 500, 1000, 2000, and 4000 Hz was performed in only one ear of each subject, chosen according to the subject's perception of his/her “better ear” or at random, in the absence of reference.

To detect and record the CAEP, the HEARLab^®^ system was used, which has a module that enables the assessment of cortical electrophysiological threshold (cortical tone evaluation).

The assessment of cortical thresholds for tone burst followed the following protocol, depending on the assessment group: 1) For Group C, adults with normal hearing, the initial intensity of 70 dB HL was used; the response was recorded and then decreased to 20 dB HL. When there was also cortical response at that intensity, descending intensities were used in steps of 10 dB and ascending intensities of 5 dB up to the minimum threshold of automatic response detection (minimum intensity). 2) For Group S, adults with hearing impairment, the initial intensity of 70 dB HL was also used. When there was also a cortical response at that intensity, descending intensities in steps of 10 dB and ascending intensities of 5 dB were used up to the minimum threshold of automatic response detection, as in Group C. However, if no response was detected in the initial intensity of 70 dB HL, the assessment survey was performed in increments of intensity in steps of 10 dB and descending of 5 dB up to the minimum threshold of cortical response detection. The detected thresholds were termed CET.

The automatic detection of the presence and respective cortical response threshold to acoustic stimulation takes into account the statistical *p*-value in an exclusive software provided with the equipment. The lower the *p*-value, the greater the probability that the recorded wave is related to the sound stimulus. A *p*-value of 0.05 is the threshold considered by the equipment and examiner for decision-making regarding the presence or absence of cortical response; that is, this threshold means that there would be only a 5% chance for this equipment to record a response as false-positive.

### Statistical analysis

For statistical analysis, central tendency measurements, Pearson's correlation, and linear regression were used.

In all tests, a 0.05 significance level (or 5%) was used to reject the null hypothesis.

## Results

The final sample of this study included the participation of 52 adult subjects (total), divided into two groups, Group C and Group S, composed of individuals with normal hearing and hearing loss, respectively. In Group C, 31 adults were evaluated: 23 (74.2%) females and eight (25.8%) males; mean age of 23.7 years (SD = 5.2 years), with mean tritonal audiometric thresholds of 2.1 dB HL (SD = 2.9 dB HL). In Group S, 21 adults participated: 15 (71.4%) females and six (28.6%) males, mean age of 48.9 years (SD = 7.2 years), with mean tritonal valuesat 500, 1000, and 2000 Hz of 58 dB HL (SD = 12 dB HL; Table 1).

**Table 1:** Demographic data of subjects in study and control groups.

Group	N ears (participants)	Age (years)	Auditory threshold (dB HL)
Study	21 (21)	48.9 (±7.2)	58 (±12)
Control	31 (31)	23.7 (± 5.2)	2.1 (±2.9)

Descriptive measures of AT, BT, and CET for the frequencies of 500, 1000, 2000, and 4000 Hz are shown in Table 2 for Group C and in Table 3 for Group S.

**Table 2:** Descriptive measures of auditory, electrophysiological, and behavioral thresholds obtained for control group by frequency.

Control group												
	500 Hz			1000 Hz			2000 Hz			4000 Hz		
	AT	CET	BT	AT	CET	BT	AT	CET	BT	AT	CET	BT
Mean	3.39	18.23	9.35	1.45	15.9	5.65	1.45	15.97	3.71	2.58	17	3.55
Median	5	20	10	0	15	5	0	15	0	0	20	5
SD	3.2	6.2	5.4	2.6	6.8	4.7	2.9	7.1	5	4	6.4	3.6
CI	2.4	4.49	3.9	1.94	5.05	3.51	2.16	5.22	3.78	3.4	4.72	2.71

SD, standard deviation; CI, confidence interval; AT, auditory threshold; CET, cortical electrophysiological threshold; BT, behavioral threshold.

**Table 3:** Descriptive measures of auditory, electrophysiological, and behavioral thresholds for the study group by frequency.

Study group												
	500 Hz			1000 Hz			2000 Hz			4000 Hz		
	AT	CET	BT	AT	CET	BT	AT	CET	BT	AT	CET	BT
Mean	49.2	57.8	55.2	56.1	65.7	60	57.1	63.1	60	55	62.1	57.8
Median	50	60	60	60	65	60	60	60	60	55	65	60
SD	13.8	6.2	13.8	12.9	9.7	12.9	11.3	11.2	9.8	11	10.3	11.2
CI	12.57	4.59	12.59	11.78	8.91	11.78	10.34	10.23	8.98	10.1	9.39	5.24

SD, standard deviation; CI, confidence interval; AT, auditory threshold; CET, cortical electrophysiological threshold; BT, behavioral threshold.

At all frequencies, it could be observed that CET had higher thresholds compared to AT. On average, the differences between these thresholds were 8.6, 9.6, 6.0, and 7.1 dB for frequencies of 500 Hz, 1000 Hz, 2000 Hz, and 4000 Hz, respectively, in Group S. For Group C, the mean differences were 14.8, 14.5, 14.5, and 14.8 dB, respectively, for the same frequencies.

The linear regression analyzes performed between AT for pure tone (“gold standard”) and BT for specific-frequency stimuli (tone bursts) showed that these thresholds are strongly correlated to all frequencies (*r*^2^ ≥ 0.7) in both groups. Thus, it was decided to correlate the behavioral and electrophysiological thresholds.

**Figure 1:**
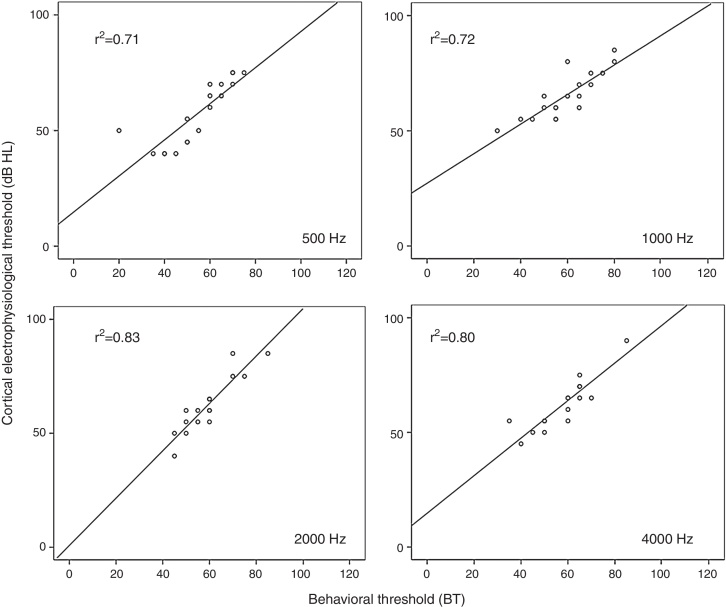
Linear scatter plots of the group with hearing loss. Behavioral threshold (BT) × cortical electrophysiological threshold (CET) to tone burst at 500, 1000, 2000, and 4000 Hz.

In Group C, the correlation between CET and BT was poor. However, in Group S, the strong correlation between the two measures is evidenced by *r*^2^ = 0.71; 0.72; 0.83, and 0.80 for all studied frequencies of 500 Hz, 1000 Hz, 2000 Hz, and 4000 Hz, respectively. Fig. 1 shows the scatter plots of Group S, for the four audiometric frequencies separately with CET on the vertical axis and BT on the horizontal axis.

## Discussion

This study assessed and compared the behavioral hearing thresholds obtained through pure tones (“gold standard” – AT) and through specific frequencies (tone burst – BT) of all individuals in the sample, for both groups with loss hearing and with normal hearing. Due to the strong correlation between the two thresholds for all frequencies (*r*^2^ ≥ 0.7); regardless of the group, we chose in this study to correlate CET with BT.

Given that the criteria for inclusion in the sample of both groups assumed the presence of symmetrical tonal thresholds bilaterally and aiming to reduce the duration of the test, the authors chose to assess the BT and CET at frequencies of 500, 1000, 2000, and 4000 Hz only in one ear of each subject, chosen according to their perception of their “better ear” or, in the absence of reference, randomly. In the study by Frizzo et al.,^16^ there were no statistically significant differences between the cerebral hemispheres that could hinder the CAEP assessment on either ear. Thus, the evaluation protocol and measurement of behavioral and electrophysiological thresholds had an approximate duration of 60 min per subject.

The technical protocol used was that suggested by the manufacturer of the HEARLab System^®^ (Frye Electronics, United States). The impedance of electrodes remained balanced and did not exceed 5 kΩ. The sound stimuli presented through insert earphones were tone bursts with 150 μs duration, although Lightfoot^17^ has reported that the use of 10–20 μs duration stimuli are sufficient to capture the response, except when it is near the subject's cortical threshold.

In the present study, subject alertness during the evaluation was controlled and maintained. Näätänen^18^ warned of the influence of drowsiness effects on variability of cortical responses. Unlike the short-latency electrophysiological responses, which require quality control and muscle relaxation during the capture of CAEP, the subject simply needs to stay awake and consciously alert. This can be achieved by asking him/her to remain in the sitting position, while entertained with the images of a video without sound, for example. The difference of this system over its currently available counterparts, is that it is a device with advanced and differentiated technologies that, in addition to reducing the registration noise and interference, provides residual noise control measures and features increased-sensitivity electrodes for the capture of responses.^3^

The automatic CAEP responses detection equipment aims to facilitate the examiner's task of subjectively interpreting the electrophysiological waves based solely on a visual analysis for the presence or absence of cortical response to acoustic stimuli. The statistical method used in the equipment was shown in previous studies to be able to detect cortical responses with combined sensitivity and specificity equal to or greater than that achieved by experienced examiners,^3^, ^9^ results that are supported in the present study.

In Group S, the mean differences between CET and BT were 7.8 dB. The values found in this study are slightly higher than those reported in a preliminary study by Van Dun et al.,^11^ whose differences for the same frequencies ranged from 3.4 to 5.9 dB. In a study by this same author,^14^ investigating CAEP thresholds in 34 adults with hearing loss, the CET were on average 10 dB higher (SD = 10 dB) than the BT, similar results to the present study.

Table 4 presents an overview of the studies that estimated the auditory threshold in adult subjects with hearing loss, reporting differences between cortical electrophysiological and behavioral thresholds ranging from 9 to 14 dB, with standard deviation of 5–14 dB.

**Table 4:** Overview of studies performed with adults with hearing loss for behavioral auditory threshold assessment using CAEP.

Study	Ears (participants)	Mean age (range) years	Hearing loss dB HL	Dur (ms))	PR (s)	N stimulus	Electrophysiological behavioral difference (dB)				
							500 Hz	1000 Hz	2000 Hz	4000 Hz	Median
Beagley and Kellogg (1969)^20^	36 (36)	32 (18–52)	n/ref	25	1.25	60	3 ± 6	1 ± 6	4 ± 7		3 ± 5
Coles and Mason (1984)^21^	129 (129) ML	n/ref	n/ref	200	1.5	64	0 ± 10	−1 ± 6	−1 ± 11	−2 ± 7	
Hoth (1993)^22^	21 (21)	18–78	10–100	500	2.5	50	Objective visual detection				5 ± 12
											−2 ± 11
Prasher et al. (1993)^23^	62 (62) PAIR	55 ± 10 (34–78)	28 ± 17^a^	200	1.0	s/ref		0 ± 11		1 ± 10	
			53 ± 22^b^								
	27 (27) Meniere	59 ± 10 (39–73)	49 ± 23^a^					2 ± 8		1 ± 8	
			58 ± 15^b^								
Richards et al. (1996)^24^	982 (500) ML	55 ± 8	5–100	100	2.0	s/ref	1 ± 5	1 ± 4	2 ± 5	0 ± 5	1 ± 5
Tsui et al. (2002)^25^	408 (204) ML	36–74	10–120	200	0.8	64		2 ± 11	1 ± 9		
Tomlin et al. (2006)^26^	30 (30)	67 (36–91)	>20	100	1.4	60	9 ± 7			14 ± 14	
Yeung and Wong (2007)^27^	44 (34)	23–69	30–55				7 ± 8	8 ± 5	5 ± 10	3 ± 14	
			60–85				6 ± 7	9 ± 8	8 ± 9	3 ± 19	
			90+				−2 ± 5	2 ± 5	6 ± 7	9 ± 10	
Van Dun et al. (2015)^14^	66 (34)	71 ± 9 (43–89)	50–18	40	1.175	120	11 ± 8	11 ± 9	10 ± 12	9 ± 11	10 ± 10
Present study	21 (21)	48.9 ± 7.2	58 ± 12	40	1.175	50–120	8 ± 9	9 ± 7	6 ± 7	7 ± 8	8 ± 8

Dur, duration; n/ref, no reference; PR, presentation rate; ML, medico-legal; NIHL, noise-induced hearing loss.

a 1000 Hz.

b 2000 Hz. *Note*: Studies involving several individuals (participants with normal hearing and hearing loss) that could not be separated were not included. All CAEP were evaluated by visual inspection of the responses, except in the studies by Hoth,^22^ Van Dun et al.,^14^ and the present study. This table structure is similar to Table 11.1 by Picton.^28^ All studies, except those by Beagley and Kellogg,^20^ Coles and Mason,^21^ and Rickards et al.,^24^ defined threshold as the lowest intensity level at which a response could be identified. The thresholds by Beagley and Kellogg^20^ were further reduced by 2.5 dB. Coles and Mason^21^ considered 5 dB the best estimate threshold. Rickards et al.^24^ considered the CAEP lowest intensity detection or 5 dB reduction, depending on the used criterion. Translation of the original Table (Van Dun et al.),^14^ authorized by the authors.

In Group C, the differences between thresholds were higher (mean of 14.5 dB) for the same frequencies studied. The mean difference between the present study thresholds was similar to that found in the study by Lightfoot and Kennedy.^19^ They evaluated 24 adult subjects with normal hearing and concluded that 94% of the sample thresholds were estimated with a difference ≤15 dB and 80% could have their thresholds estimated with a difference ≤10 dB. Those authors reported that, although the mean difference between the thresholds was between 5 and 10 dB in most of the sample, it was found that in a small subsample (7%) the differences between the thresholds were up to 20 dB higher for BT. Van Dun et al.^14^ studying individuals with hearing loss, also reported the presence of 4% of what they called “out of the curve” differences, referring to the subjects that presented differences between thresholds up to 30 dB, with cortical threshold CET always higher than BT. In the present study, this small group was also present in 4% of records with differences up to 30 dB. Paradoxically, cortical threshold responses, comparatively lower than those recorded for BTs up to 10 dB were also observed in 2.4% of records.

When comparing the mean detection and response thresholds of CAEP between the groups of subjects with normal hearing and subjects with hearing loss, the difference between BT and CET was higher in the Group C. This difference between groups was also reported in the study by Golding et al.^3^ A possible explanation for the subjects in the Group S to record CET closer to the BT or with a minor sensation level (SL) appears be based on the potential impact of the recruitment phenomenon on those subjects with sensorineural hearing loss, which would increase the amplitude of CAEP response at lower or weaker SLs.^9^, ^10^

Given the findings of this study, the CAEP obtained through automatic response analysis equipment was shown to be a viable test to estimate the auditory threshold in adults with hearing loss.

Complementary studies using the automatic cortical response threshold analysis equipment would be of great clinical relevance to establish correction factors for the assessment of hearing thresholds, as well as the evaluation of different populations.

## Conclusion

The results of this study indicated a strong correlation between behavioral thresholds and cortical electrophysiological thresholds for frequencies of 500, 1000, 2000, and 4000 Hz in adults with hearing loss.

## Conflicts of interest

The authors declare no conflicts of interest.
